# Cell Therapy: Effect of Locally Injected Mesenchymal Stromal Cells Derived from Bone Marrow or Adipose Tissue on Bone Regeneration of Rat Calvarial Defects

**DOI:** 10.1038/s41598-019-50067-6

**Published:** 2019-09-17

**Authors:** Gileade P. Freitas, Helena B. Lopes, Alann T. P. Souza, Paula G. F. P. Oliveira, Adriana L. G. Almeida, Lucas E. B. Souza, Paulo G. Coelho, Marcio M. Beloti, Adalberto L. Rosa

**Affiliations:** 1 Bone Research Lab, School of Dentistry of Ribeirão Preto, University of São Paulo, São Paulo, SP Brazil; 2 Hemotherapy Center of Ribeirão Preto, University of São Paulo, São Paulo, SP Brazil; 3 Department of Biomaterials, New York University College of Dentistry, New York, NY USA; 4 Hanjorg Wyss Department of Plastic Surgery, New York University School of Medicine, New York, NY USA

**Keywords:** Regeneration, Mesenchymal stem cells

## Abstract

Treatment of large bone defects is a challenging clinical situation that may be benefited from cell therapies based on regenerative medicine. This study was conducted to evaluate the effect of local injection of bone marrow-derived mesenchymal stromal cells (BM-MSCs) or adipose tissue-derived MSCs (AT-MSCs) on the regeneration of rat calvarial defects. BM-MSCs and AT-MSCs were characterized based on their expression of specific surface markers; cell viability was evaluated after injection with a 21-G needle. Defects measuring 5 mm that were created in rat calvaria were injected with BM-MSCs, AT-MSCs, or vehicle-phosphate-buffered saline (Control) 2 weeks post-defect creation. Cells were tracked by bioluminescence, and 4 weeks post-injection, the newly formed bone was evaluated by µCT, histology, nanoindentation, and gene expression of bone markers. BM-MSCs and AT-MSCs exhibited the characteristics of MSCs and maintained their viability after passing through the 21-G needle. Injection of both BM-MSCs and AT-MSCs resulted in increased bone formation compared to that in Control and with similar mechanical properties as those of native bone. The expression of genes associated with bone formation was higher in the newly formed bone induced by BM-MSCs, whereas the expression of genes involved in bone resorption was higher in the AT-MSC group. Cell therapy based on local injection of BM-MSCs or AT-MSCs is effective in delivering cells that induced a significant improvement in bone healing. Despite differences observed in molecular cues between BM-MSCs and AT-MSCs, both cells had the ability to induce bone tissue formation at comparable amounts and properties. These results may drive new cell therapy approaches toward complete bone regeneration.

## Introduction

Bone tissue has a higher capacity to regenerate when injured by trauma, infections, or neoplasia. However, in some cases, the extent of damage may exceed the inherent tissue regeneration capability, as reported in approximately 5% of traumatic fractures^1^. These situations require the use of additional treatments, and grafts of autogenous, allogeneic, or alloplastic natures are often used in an attempt to achieve complete regeneration of extensive bone defects. Although grafting has a relative clinical success, it is associated with problems such as morbidity due to a second surgical site and the possibility of disease transmission^2,3^. Therefore, the use of cells through two different approaches, tissue engineering and cell therapy, has attracted the attention of several research groups. Tissue engineering combines cells with biomaterials, whereas cell therapy is based only on cells injected either locally or systemically^4–6^.

In the past decade, our research group had evaluated tissue engineering strategies using PLGA/CaP, gelatin sponge, bioglass, and a polymeric membrane as carriers for bone marrow-derived mesenchymal stromal cells or osteoblasts to treat calvarial defects^7–10^. In general, the combination of biomaterials with cells resulted in increased bone formation; however, none of those studies demonstrated complete regeneration of the bone tissue. At least in part, this is due to the unpredictable biological behaviour of the carriers that can be degraded very quickly or remained too long but in either way disturbing the process of bone regeneration. Therefore, in this study, we intended to evaluate the effectiveness of cell therapy in inducing bone repair.

The first study reporting about the intravenous infusion of cells isolated from bone marrow in patients with osteogenesis imperfecta pioneered the treatment of skeletal diseases through cell therapy^11^. The treatment of unconsolidated fractures using bone marrow aspirates directly injected into the fracture site was found to be successful in 53 of 60 patients, and the local injection of osteoblasts also accelerated bone repair in long bone fractures^12–14^. Preclinical investigation has demonstrated that treatment with mesenchymal stromal cells derived from bone marrow (BM-MSCs) induced bone formation in distraction osteogenesis, in cases of femoral head osteonecrosis, and in bone defects^15–18^. Previous research has also reported about the treatment of bone defects immediately after their creation, exposing the delivered cells to a large number of cytokines, including proinflammatory IL-1β and TNF-α that were released as a part of the host immune response to the surgical procedure^19^. Although promising results were achieved, such an experimental design does not mimic clinical scenarios, wherein it is required to treat pre-existent defects that are more challenging to repair due to the presence of connective tissue. In an attempt to evaluate bone regeneration in more challenging conditions, we developed a model to investigate the use of cell therapy to treat bone defects where cells are injected 2 weeks after the creation of the defects. Using this model, osteoblasts were injected into the bone defects either on a preimplanted membrane or directly into the bone defects^10,20^. In both studies, the extent of bone formation was higher with the use of cells; however, the defects were not fully regenerated, thereby warranting alternative approaches such as the application of undifferentiated MSCs.

The conventional source of MSCs is the bone marrow due to its well-known role in the repair of bone fractures^21,22^. These cells are often obtained from the crest of the ilium, acetabulum, or femur^23^. However, due to the invasiveness of the procedure, the low incidence of MSCs (1 to 10^5^ cells), and the decline in both their proliferation and differentiation potential with the increase in senescence, other tissues such as the epithelium, muscle, fat, and articular cartilage have been investigated as cell sources^24–28^. Hence, MSCs harvested from the adipose tissue (AT-MSCs) have received attention as they can be obtained using less invasive procedures while presenting a higher number of cells^29^. In addition, there is evidence indicating that AT-MSCs enhanced angiogenesis comparable to that of BM-MSCs^30^. Moreover, some studies have demonstrated the capacity of AT-MSCs to stimulate bone repair in different animal models such as femoral fracture and critical size calvarial defects^31–33^. Although the potential of MSCs derived from both sources to induce bone formation has been demonstrated, the comparison between BM-MSCs and AT-MSCs as cell therapy to regenerate bone tissue remains underexplored in the current literature.

Considering the above-described scenario, the aim of this study was to evaluate the efficacy of cell therapy in bone regeneration. We hypothesized that both BM-MSCs and AT-MSCs when locally injected can induce bone regeneration in large pre-existent bone defects. To evaluate this hypothesis, we created 5-mm rat calvarial defects and administered local injections of either BM-MSCs or AT-MSCs 2 weeks after the creation of the defects in an attempt to mimic a treatment of inducing bone repair in challenging clinical situations.

## Methods

### Animals

 In all experiments, we used 28-day-old male Wistar rats weighing 250 g, which were maintained in the animal facility with constant temperature (22 °C ± 2 °C), free access to water and food, and a standard light/dark cycle of 12-h light followed by 12-h dark.

### Harvesting and expansion of BM-MSCs and AT-MSCs

Cells were harvested as previously described^10^. The BM-MSCs were harvested from the femur medullary canals and cultivated in growth medium that is alpha minimum essential medium (α-MEM, Gibco-Invitrogen, Grand Island, NY, USA) supplemented with 10% fetal bovine serum (Gibco-Invitrogen), 50 μg/ml gentamicin (Gibco-Invitrogen), and 0.3 μg/ml fungizone (Gibco-Invitrogen). The AT-MSCs were isolated from the inguinal adipose tissue using 0.075% type II collagenase (Gibco-Invitrogen) at 37 °C for 40 min. The cells were centrifuged, the floating adipocytes were removed, and the pellet was resuspended in a growth medium. Both BM-MSCs and AT-MSCs were cultured in the growth medium in 75-cm^2^ flasks for 10 days. During the entire culture time, the cells were maintained at 37 °C in a humidified atmosphere of 95% air and 5% CO_2_, and the medium was changed every other day.

### Characterization of BM-MSCs and AT-MSCs by flow cytometry

On day 10, both BM-MSCs and AT-MSCs were detached from the polystyrene flask using a solution containing 0.25% trypsin (Gibco-Invitrogen), 1.3 mg/ml type II collagenase (Gibco-Invitrogen), and 1 mM ethylenediaminetetraacetic acid (EDTA; Gibco-Invitrogen). The number of cells was adjusted to 2 × 10^5^/tube and separately incubated for 30 min at room temperature with the following monoclonal anti-rat antibodies: anti-CD29, -CD31, -CD34, -CD45, and -CD90 (BD Biosciences, San Jose, CA, USA). The cells were then washed in 2 ml of phosphate buffered saline (PBS, Gibco-Invitrogen)/Tween-20 (Sigma-Aldrich, St Louis, MO, USA) and centrifuged at 1500 rpm for 5 min. The supernatant was discarded, and the cells were subjected to another wash with PBS (Gibco-Invitrogen). After discarding the supernatant, 0.5 ml of formaldehyde solution (Merck, Germany) diluted to 1% in PBS was added. Flow cytometry was carried out on the FACSCanto™ system (BD Biosciences).

### Evaluation of cell viability: plastic pipette tips versus 21-gage stainless steel needle

To evaluate whether passing the cells through a 21-gage stainless steel needle (21-G) could affect cell viability, a BM-MSC suspension containing 5 × 10^6^ cells was plated in a 24-well plate containing 1 ml of 10% MEM using 200-µl plastic pipette tips (Control) or 21-G needles. After 6, 24, and 48 h of culture, trypan blue (Sigma-Aldrich) was added into the cell suspension (1:1), and viable cells were evaluated by cell counting using a hemocytometer (Hausser Scientific, Horsham, PA, USA). Data were expressed as the percentage of Control.

### Surgical procedure

The rats were anesthetized, and the surgical procedure was performed as previously described^8–10^. Briefly, after anesthetizing the rats with a solution containing ketamine (7 mg/100 g body weight, Agener União, Embu-Guaçu, SP, Brazil) and xylazine (0.6 mg/100 g body weight, Calier, Juatuba, MG, Brazil), the parietal bone was exposed by a sagittal incision in the scalp and unilateral calvarial defects of 5-mm diameter were created using a trephine drill (Neodent, Curitiba, PR, Brazil) under saline solution irrigation. Then, the tissue was sutured with a 4.0 silk thread and the limits of the bone defects were drawn on the skin with permanent markers allowing their location for later cell injection. After the surgery, each animal was medicated with an intramuscular injection of 0.1 ml/100 g body weight of pentabiotic (Fort Dodge, Campinas, SP, Brazil) and 0.2 ml/100 g Banamine® (Schering-Plough, Kenilworth, Nova Jersey, USA) for pain management.

### BM-MSCs and AT-MSCs expressing luciferase

To track the presence of cells in the bone defects, bioluminescence imaging of luciferase-expressing BM-MSCs and AT-MSCs was conducted as described elsewhere^10^. Briefly, at 80% confluence, both BM-MSCs and AT-MSCs at the first passage were infected with virions containing the bicistronic lentiviral vector pMSCSV-Luc2-T2A-Puro (kindly provided by Dr. Deivid de Carvalho Rodrigues) that encodes the bioluminescent reporter Luc and the resistance marker puromycin N-acetyltransferase. After 48 h, the transduced cells were maintained in a medium containing 1 µg/ml puromycin for 6 days to sort out BM-MSC-Luc and AT-MSC-Luc expressing cells that were further expanded for 10 days and deprived of fetal bovine serum for the final 24 h before being injected to avoid the risk of foreign body reaction.

### Cell injection

After 2 weeks of the defect creation, the animals were anesthetized as described above and the calvarial defects were administered a local injection of 5 × 10^6^ cells in 50 µl of vehicle-PBS or 50 µl of only PBS (Control) through a 21-G stainless steel needle coupled to a micropipette. The needle was inserted tangent to the skullcap with the tip of the needle near the centre of the bone defect and the needle bevel facing ventrally. BM-MSC-Luc and AT-MSC-Luc were injected to track the cells, and BM-MSCs and AT-MSCs were injected to evaluate bone formation.

### Cell tracking

Bioluminescence imaging of BM-MSC-Luc or AT-MSC-Luc was conducted using the Lumina *in vivo* system equipment (IVIS, Caliper Life Sciences, Hopkinton, MA, USA) from day 4 to 14 after the injection. For image acquisition, the animals were anesthetized with 2% isoflurane and a subcutaneous injection of a solution containing 100 µl of d-luciferin (Sigma-Aldrich) at a concentration of 30 mg/ml was administered in the dorsal region of the animals. The rats, under continuous exposure to 2% isoflurane, were positioned into the IVIS camera box, the region of interest was manually determined around the bioluminescent signal, and the intensity was detected as photons/s.

### Bone formation

After 4 weeks of BM-MSC or AT-MSC injection, the animals were euthanized and the calvarias were harvested and fixed in 4% paraformaldehyde. Bone formation was evaluated by microcomputed tomography (µCT), histology, and nanoindentation. The gene expression of bone remodelling markers was also assessed.

#### µCT analysis

The calvarias were submitted to µCT analysis as previously described^8–10^. The volume of interest (VOI) selected to determine the borders and limits of the defects was 5 mm in diameter and 0.5 mm in thickness. After delimitation of the VOI, the bone segmentation within the defect was defined between 85 and 255 in a gray histogram from 0 to 255. The 3D Ctan software (Bruker-Skyscan) was used to determine the following morphometric parameters: bone volume, percentage of bone volume, bone surface, trabecular thickness, trabecular number, and trabecular separation, as previously described^34^.

#### Histological analysis

After the µCT scanning, the samples were prepared and sectioned as previously described^8–10^. Histological sections were prepared using the Exakt Grinding System (Exakt) and stained with Stevenel’s blue and Alizarin red. The histological description of the tissues was based on light microscopy images obtained using a Leica DMLB light microscope (Leica, Bensheim, Germany).

#### Nanoindentation assay

The elastic modulus and the hardness of the formed bone were evaluated using a TI 950 nanoindenter (Hysitron, Minneapolis, Minnesota, USA). The calvarial bone harvested during the defect creation was used as the control (native bone). For this purpose, nonstained nondecalcified histological slides were polished with the diamond suspension, ranging from 1 to 9 μm (Buehler, Lake Bluff, IL, USA), and hydrated for 24 h. The bone tissue was analyzed by imaging under a light microscope coupled to the TI 950 nanoindenter. In total, an average of 25 indentations were performed on the bone with the nanoindenter using three slides per group (n = 3). The charge profile was developed with a peak of 300 μN and a rate of 60 μN/s, followed by a charge time of 10 s and a discharge time of 2 s. The extended loading period allows the bone a relaxation for a larger linear response, regardless of the effect of the creep of the tissue engaging the discharge portion. Then, from each indentation data, a load–displacement curve was acquired as described elsewhere^35^. From each of the generated load–displacement curves, the elastic modulus (GPa) and the hardness (GPa) of the cortical bone tissue were computed using the Hysitron TriboScan software^36,37^.

#### Gene expression of bone remodeling markers

Quantitative real-time polymerase chain reaction (PCR) was performed to evaluate the gene expression of runt-related transcription factor 2 (*Runx2*), osterix (*Sp7*), alkaline phosphatase (*Alp*), osteocalcin (*Oc*), bone morphogenetic protein 4 (*Bmp4*), osteopontin (*Opn*), receptor activator of nuclear factor-kappa B ligand (*Rankl*), osteoprotegerin (*Opg*), and tartrate-resistant acid phosphatase (*Trap*). The bone formed in the defects injected with BM-MSCs or AT-MSCs was carefully removed using a trephine under saline irrigation to avoid RNA degradation. For nanoindentation assays, the native bone was used as the control. Total RNA was obtained from crushed bone fragments using Trizol reagent and reverse-transcribed into complementary DNA (cDNA) according to the manufacturer’s recommendations (Life Technologies-Invitrogen, Carlsbad, CA, USA), as previously described^38^. The concentration and purity of RNA samples were determined using a GeneQuant® spectrophotometer (GE Healthcare, Buckinghamshire, UK), and integrity was evaluated using the 2100 Bioanalyzer (Agilent Technologies, Stockport, UK). cDNA was synthesized using 1 µg of RNA through a reverse transcription reaction using a high-capacity cDNA reverse transcription kit (Applied Biosystems, Foster City, CA, USA), according to the manufacturer’s instructions. Real-time PCR was carried out in triplicate (n = 3) and performed in the StepOnePlus Real-Time PCR System (Thermo Fisher Scientific, Waltham, MA, USA) with the following thermal cycling conditions: 50 °C (2 min), 95 °C (20 s), and 40 cycles of 95 °C (3 s) and 60 °C (30 s) in a 10-μl reaction volume using 5 µl of TaqMan universal PCR master mix AmpErase UNG 2X (Life Technologies-Invitrogen), 0.5 µl of TaqMan probes (Life Technologies-Invitrogen), and 11.25 µg of cDNA. The relative gene expression was calculated using β-actin as the housekeeping gene, and the actual changes were relative to the gene expression of the native bone.

### Statistical analyses

The results of cell viability (n = 5), morphometric parameters (n = 12), nanoindentation (n = 3 for each parameter), and real-time PCR (n = 3 for each gene) analyses were compared by one-way analysis of variance (ANOVA), followed by Tukey’s test wherever appropriate. The results of cell tracking (n = 3) analysis were compared by two-way ANOVA, followed by Tukey’s test wherever appropriate. For all experiments, the level of significance was established at p ≤ 0.05. Numerical data were expressed as mean ± standard deviation.

### Ethical approval

All procedures performed on animals were conducted in accordance with ethical standards of the international, national, and/or institutional animal care guidelines. The Committee of Ethics in Animal Research of the School of Dentistry of Ribeirão Preto, University of São Paulo, reviewed and approved all animal procedures we have done here(# 2015.1.191.58.9).

## Results

### Characterization of MSCs

**Figure 1:**
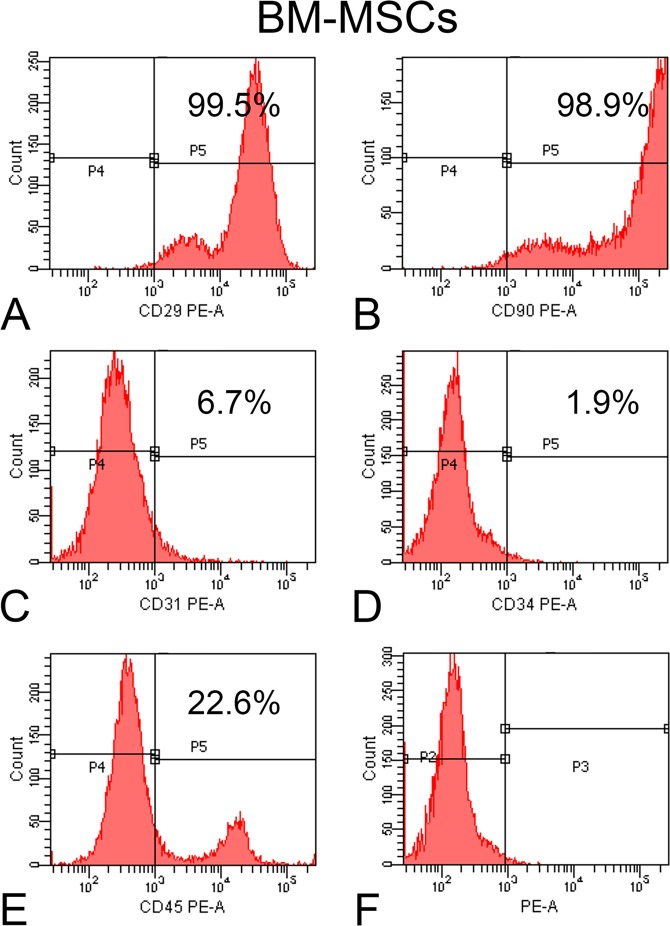
Flow cytometry of bone marrow-derived mesenchymal stromal cells (BM-MSCs). Histograms show the high expression of CD29 (**A**) and CD90 (**B**) and the low expression of CD31 (**C**), CD34 (**D**) and CD45 (**E**) after incubation with the respective antibodies as well as nonlabeled cells (**F**).

**Figure 2:**
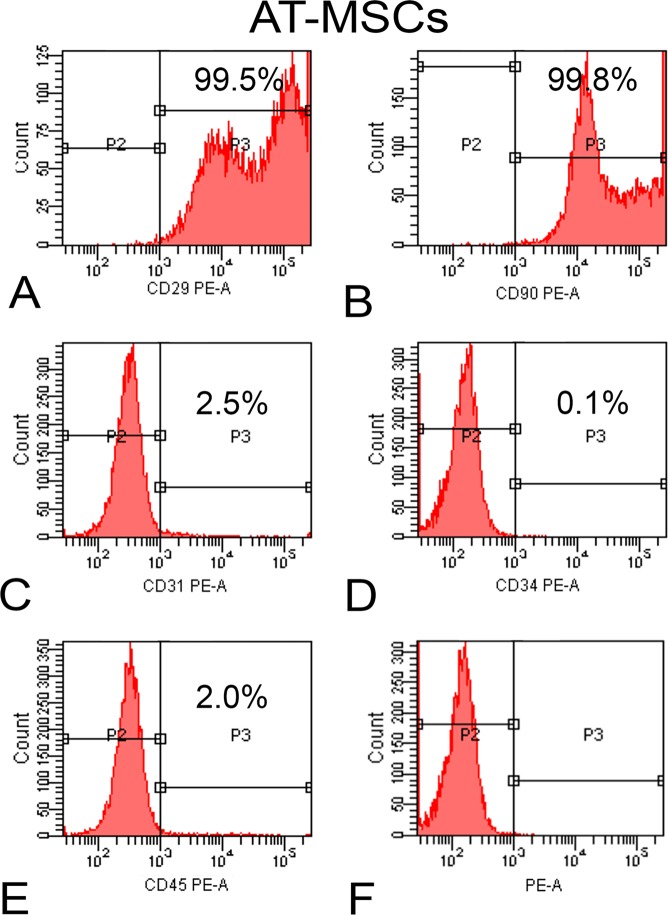
Flow cytometry of adipose tissue-derived mesenchymal stromal cells (AT-MSCs). Histograms show the high expression of CD29 (**A**) and CD90 (**B**) and the low expression of CD31 (**C**), CD34 (**D**), and CD45 (**E**) after incubation with the respective antibodies as well as nonlabeled cells (**F**).

At 10 days of cell culture, the BM-MSCs (Fig. 1A–F) and AT-MSCs (Fig. 2A–F) exhibited a high percentage of expression of the surface markers CD29 (99.5% for both cells) and CD90 (98.9% and 99.8%, respectively), the characteristics of MSCs, whereas the expression percentages of CD31 (6.7% and 2.5%, respectively), CD34 (1.9% and 0.1%, respectively), and CD45 (22.6% and 2.0%, respectively) were less, which are recognized as hematopoietic markers. Hence, the evaluated surface markers confirmed that the majority of both cell populations are MSCs.

### Evaluation of cell viability: plastic pipette tips versus 21-G stainless steel needle

There was no significant effect on cell viability when the cells were passed through either a plastic pipette or a 21-G needle at any time point of the culture after the injections (6 h, p = 0.27; 24 h, p = 0.97; and 48 h, p = 0.65). The results are represented as the percentage of viable cells (Table 1). Then, the use of the 21-G needle was validated to inject cells into the bone defects without affecting their viabilities.

**Table 1:** Percentage of viable cells after plating 5 × 10^6^ bone marrow-derived mesenchymal stromal cells (BM-MSCs) using plastic pipette tips or 21-G stainless steel needle evaluated at 6, 24, and 48 h post-plating.

Time (h)	Plastic pipette (% viable cells)	21-G needle (% viable cells)	p value
6	100	86.95	0.274
24	100	99.1	0.974
48	100	90.9	0.659

### Cell tracking

**Figure 3:**
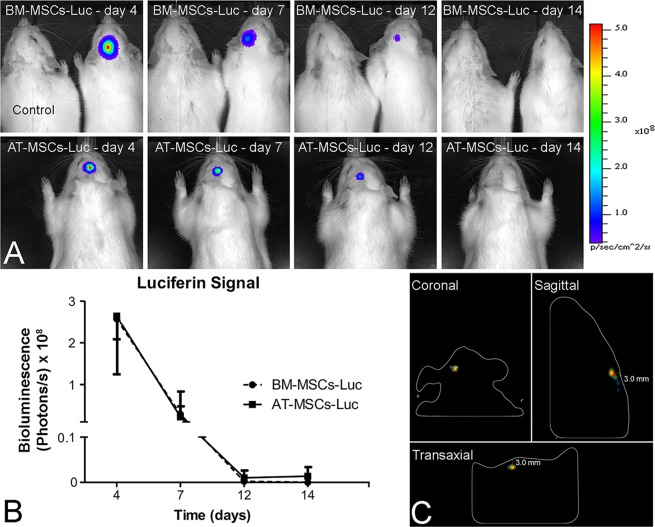
Tracking of bone marrow-derived mesenchymal stromal cells and adipose tissue-derived mesenchymal stromal cells transduced to express luciferase (BM-MSC-Luc and AT-MSC-Luc, respectively) and injected into rat calvarial bone defects. The presence of cells from both groups was noticed in the well-defined area of bone defects after injection. (**A**) The luciferin signal peaked at day 4 and gradually decreased until day 14. (**B**) Three-dimensional reconstructed IVIS images of BM-MSC-Luc injected into rat calvarial bone defects on coronal, sagittal, and transaxial sections (**C**) 4 days post-injection. Data are presented as mean ± standard deviation (n = 3). Animal on the left side (**A**): rat injected with phosphate-buffered saline (PBS) without cells (Control).

The results of cell tracking by bioluminescent analyses indicated the presence of BM-MSC-Luc and AT-MSC-Luc in the area of the bone defects after the injection (Fig. 3A). Quantification of the luciferin signal revealed no difference between BM-MSC-Luc and AT-MSC-Luc at any time point after the injection (p = 0.24). Both MSCs exhibited the same pattern of luciferin signal that peaked at day 4 after implantation and progressively declined until day 14 (Fig. 3B). The 3D reconstructions generated by the IVIS demonstrated that the cells were highly concentrated in the injected areas (Fig. 3C).

### Bone formation

**Figure 4:**
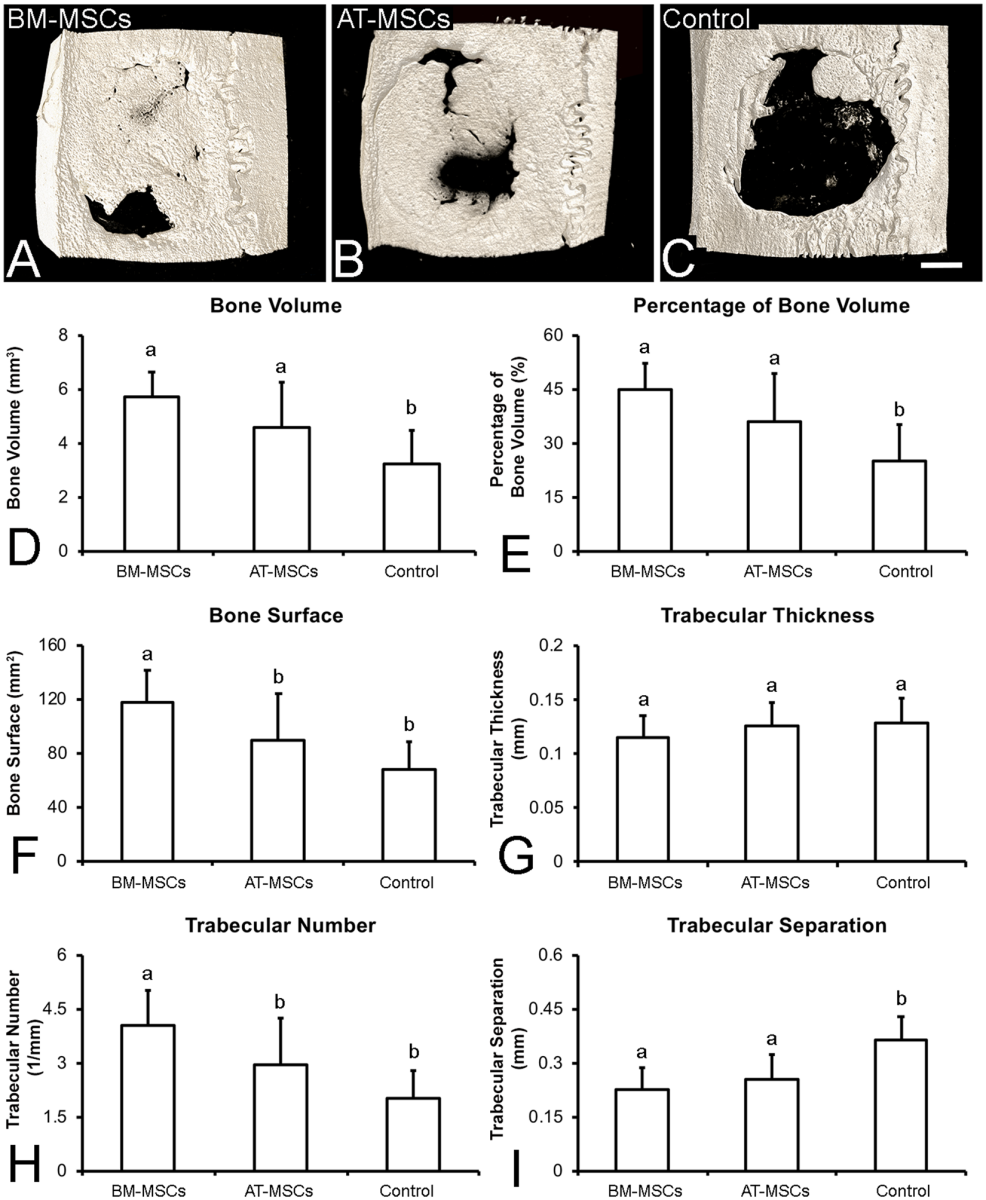
Three-dimensional reconstructed µCT images and morphometric parameters of bone formation in rat calvarial bone defects injected with bone marrow-derived mesenchymal stromal cells (BM-MSCs, **A**), adipose tissue-derived mesenchymal stromal cells (AT-MSCs, **B**), or vehicle without cells (Control, **C**) 4 weeks post-injection. The data D–I are presented as mean ± standard deviation (n = 12). Different letters indicate statistically significant differences between groups (p ≤ 0.05). Scale bar: A–C = 1.25 mm.

The 3D reconstructed µCT images revealed that at 4 weeks after cell injection, the defects treated with the injection of BM-MSCs or AT-MSCs exhibited more bone formation than Control (Fig. 4A–C). The morphometric parameters generated by the µCT analysis revealed that bone volume (Fig. 4D, p = 0.001) and percentage of bone volume (Fig. 4E, p = 0.001) were significantly higher in the calvarial defects treated with BM-MSCs or AT-MSCs than those in Control. The bone surface (Fig. 4F, p = 0.001) and the trabecular number (Fig. 4H, p = 0.001) were significantly higher in the calvarial defects treated with BM-MSCs than in those treated with AT-MSCs or in Control. Furthermore, trabecular separation (Fig. 4H, p = 0.001) was significantly lower in the calvarial defects treated with BM-MSCs or AT-MSCs than that in Control. Trabecular thickness (Fig. 4G, p = 0.30) was not affected by any treatment. Altogether, these data indicate that MSCs derived from both sources induced significant bone formation in the rat calvarial bone defects.

**Figure 5:**
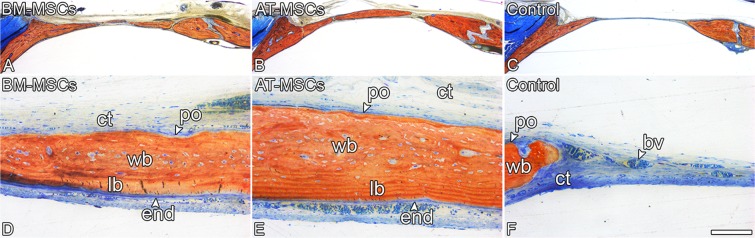
Light microscopy of rat calvarial bone defects injected with bone marrow-derived mesenchymal stromal cells (BM-MSCs), adipose tissue-derived mesenchymal stromal cells (AT-MSCs), or vehicle without cells (Control) 4 weeks post-injection. The defects treated with BM-MSCs (**A**,**D**) displayed similar bone formation compared with defects treated with AT-MSCs (**B**,**E**) and higher bone formation than defects treated with vehicle without cells. (**C**,**F**) Although new bone tissue was observed in the defects injected with BM-MSCs (**A**,**D**) or AT-MSCs (**B**,**E**), the Control defects (**C**,**F**) were filled with connective tissue. Alizarin red and Stevenel’s blue staining. bv: blood vessel, end: endosteum; lb: lamellar bone; po: periosteum; wb: woven bone; ct: connective tissue. Scale bar: A–C = 1.25 mm; D–F = 100 μm.

In general, the nondecalcified histological sections demonstrated substantial bone formation in the calvarial defects treated with BM-MSCs or AT-MSCs, whereas Control defects were primarily filled with connective tissue (Fig. 5A–F). Consistent with the µCT quantification data, the calvarial defects treated with either BM-MSCs (Fig. 5A) or AT-MSCs (Fig. 5B) exhibited comparable amounts of bone formation. Lamellar and woven bone were observed in the calvarial defects injected with both BM-MSCs (Fig. 5D) and AT-MSCs (Fig. 5E).

**Figure 6:**
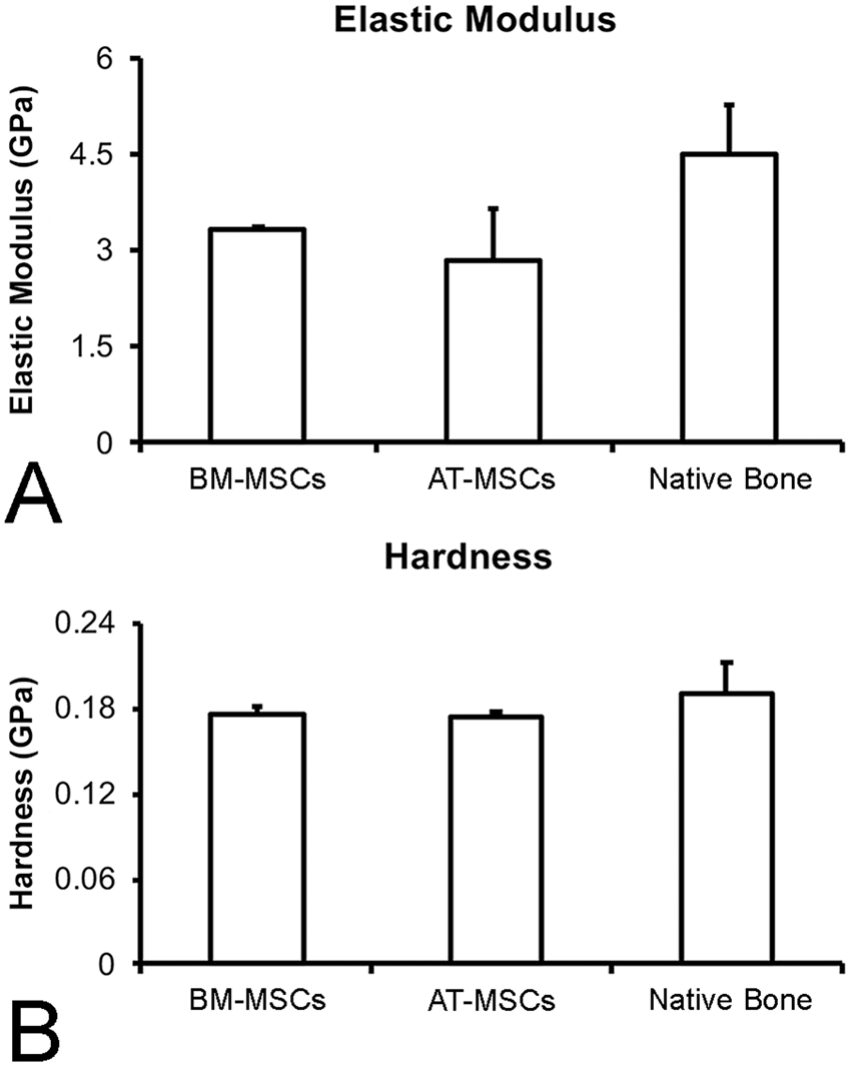
Elastic modulus (**A**) and hardness (**B**) of newly formed bone in the defects injected with bone marrow-derived mesenchymal stromal cells (BM-MSCs) or adipose tissue-derived mesenchymal stromal cells (AT-MSCs) 4 weeks post-injection compared with native bone. Data are presented as mean ± standard deviation (n = 3). No statistically significant differences were observed among the groups (p > 0.05).

The parameters generated by the nanoindentation assay, which indicate the quality of the newly formed bone, revealed no difference in the elastic modulus (Fig. 6A, p = 0.45) or hardness (Fig. 6B, p = 0.10) of the newly formed bone tissue in the defects treated with BM-MSCs or AT-MSCs compared with native bone. Moreover, regardless of the cell source, the mechanical properties of the newly formed bone tissue were similar to those of the pristine bone.

**Figure 7:**
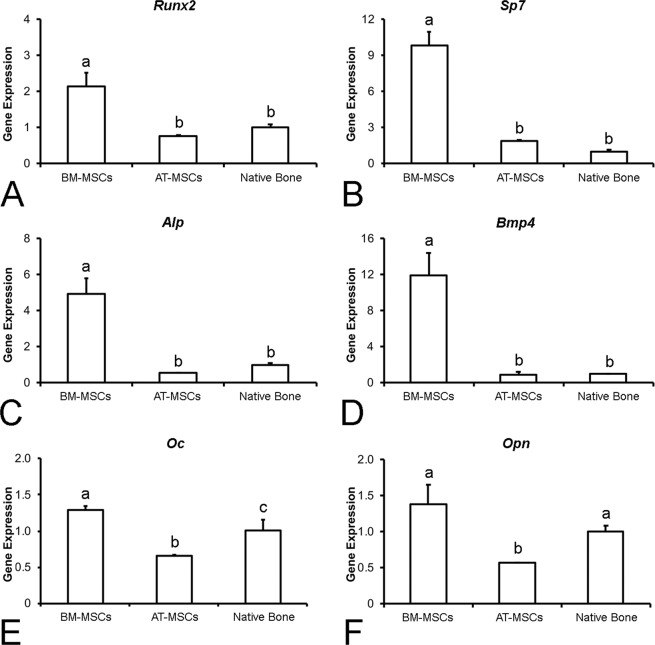
Gene expression of *Runx2* (**A**), *Sp7* (**B**), *Alp* (**C**), *Bmp4* (**D**), *Oc* (**E**), and *Opn* (**F**) of the newly formed bone in the defects injected with bone marrow-derived mesenchymal stromal cells (BM-MSCs) and adipose tissue-derived mesenchymal stromal cells (AT-MSCs), or native bone 4 weeks post-injection. The actual changes were relative to the gene expression of cells from native bone. Data are presented as mean ± standard deviation (n = 3). Different letters indicate statistically significant differences among groups (p ≤ 0.05).

**Figure 8:**
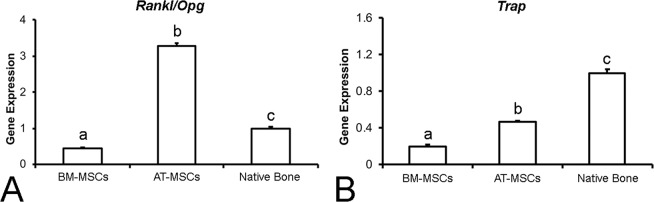
Ratio of gene expression of *Rankl*/*Opg* (**A**) and *Trap* gene expression (**B**) of the newly formed bone in the defects injected with bone marrow-derived mesenchymal stromal cells (BM-MSCs), adipose tissue-derived mesenchymal stromal cells (AT-MSCs), or native bone 4 weeks post-injection. The actual changes were relative to the gene expression of cells from native bone. Data are presented as mean ± standard deviation (n = 3). Different letters indicate statistically significant differences among groups (p ≤ 0.05).

The gene expression of *Runx2* (Fig. 7A), *Sp7* (Fig. 7B), *Alp* (Fig. 7C), and *Bmp4* (Fig. 7D) revealed a similar pattern, with higher expressions in the defects treated with BM-MSCs than in the defects treated with AT-MSCs and in the native bone; there were no significant differences between bone defects treated with AT-MSCs and native bone (p = 0.001 for all genes). The gene expression of *Oc* (Fig. 7E) was higher in the defects treated with BM-MSCs than in the defects treated with AT-MSCs and native bone, with the expression in the AT-MSC group being lower than that in the native bone (p = 0.001). The gene expression of *Opn* (Fig. 7F) was higher in the defects treated with BM-MSCs and in the native bone than in the defects treated with AT-MSCs (p = 0.003), with no significant difference between the BM-MSC-treated bone and the native bone. The ratio *Rankl*/*Opg* (Fig. 8A) was higher in the bone defects treated with AT-MSCs than in those treated with BM-MSCs and in the native bone, with a lower ratio in the BM-MSC group than in the native bone (p = 0.001). The expression of *Trap* (Fig. 8B) was higher in the native bone than in the bone defects treated with BM-MSCs and AT-MSCs, with a lower expression in the BM-MSC-treated bone than in the AT-MSC group (p = 0.001). Altogether, the gene expression data suggest that the newly formed bone induced by BM-MSCs is more predominantly in the bone-forming stage, whereas the newly formed bone induced by AT-MSCs exhibited a closer equilibrium between formation and resorption.

## Discussion

This study was designed to evaluate the effect of cell therapy with local injections of either BM-MSCs or AT-MSCs on bone formation in rat calvarial defects. For this purpose, we selected the MSCs by adherence to the tissue culture plate and characterized them based on their expression of specific surface markers. Furthermore, both cells maintained their viability after being delivered using a 21-G needle and remained in the calvarial defects for as long as 12 days. The results obtained through multiple assays support our hypothesis that the local injection of BM-MSCs or AT-MSCs can improve bone repair in large preexistent bone defects.

After harvesting, cells derived from the bone marrow of the femur and inguinal adipose tissue were cultured for 10 days to allow adherence to the tissue culture plate, and nonadherent cells were discarded through medium changes. Then, the MSCs were characterized using a panel of surface markers, which showed that these cell populations displayed a high percentage of positive cells for CD29 and CD90 and negative cells for CD31, CD34, and CD45. These data have been corroborated by previous studies reporting similar expression of these markers in MSCs harvested from Wistar rats^10,39^. The higher percentage of BM-MSCs expressing CD45 comparable to that by AT-MSCs has been attributed to the hematopoietic origin of the former cells^10^. In addition to the expression of surface markers, the MSCs derived from both sources exhibited the potential to differentiate into multiple lineages^10,39,40^.

The success of cell therapy depends on a plethora of factors, including the delivery of viable cells, cell engraftment, and the number of cells delivered. There is evidence indicating that a higher percentage of cell viability can be achieved by delivering cells through needles with shorter length and larger diameter combined with a flow rate of approximately 150 µL/min^41^. In this study, cells were directly injected into the bone defects through a short 21-G needle at a flow rate of 100 µL/min, a rate that allowed the delivery of cells with a percentage of viability similar to that obtained using plastic pipette tips. By tracking both BM-MSC-Luc and AT-MSC-Luc, the presence of cells in the calvarial bone defects was determined, and despite a marked decrease in signal from day 4 to 7, the cells were still detected for up to 12 days after the injection, which is in agreement with a previous study^10^. Despite the effectiveness of scaffolds and membranes to deliver and retain the cells in the area of interest^10,42^, here we used the strategy of injecting cells without any biomaterial 2 weeks after the defect creation, so that the newly formed connective tissue could act as a natural scaffold to retain the cells in the bone defects. In addition, the experimental model used in this study allows the injection of cells into a less hostile environment compared with inflammatory cells and cytokines that are typically encountered immediately following the defect creation^19^. There is no consensus in the literature regarding the ideal number of cells to be used in cell therapy, but it is a general idea that higher bone formation in bone defects can be achieved using a higher concentration of cells^4^. This concept has been supported by the correlation between better outcomes in cases of nonunion of fractures and a higher number of cells^12^. Based on this finding and considering the size of the MSCs on the culture plate^43^, we chose to inject 5 × 10^6^ cells/defect, which is the maximum number of cells that could be taken into a bone defect with a diameter of 5 mm and a height of 0.5 mm, which implies half a million cells per cubic millimeter of tissue. This appears to be the ideal cell concentration to optimize bone formation by local cell therapy, as this is the maximum number of cells that is possible to pack per cubic millimeter and injecting lower cell concentrations could result in reduced bone formation (Fig. S1).

Regarding bone formation, the 3D reconstructed µCT images, the morphometric parameters, and the histological sections confirmed that the MSCs directly delivered into the calvarial defects can induce more bone formation than vehicle-PBS. These findings are consistent with the bone formation induced by the injection of osteoblasts derived from newborn rat calvaria^20^. A previous study has reported the capacity of MSCs to act as a therapeutic approach due to their ability to change the host microenvironment rather than their capacity to differentiate and incorporate into the host tissue^44^. In addition, it has been shown in the cardiac muscle and corneal epithelium that the delivered cells may transfer mitochondria to the host cells, a mechanism that may contribute to the repair of damaged tissues^45–47^. Based on the morphometric indicators of bone regeneration, such as bone surface and trabecular number, the BM-MSCs demonstrated improved results compared with AT-MSCs; however, further investigation is warranted before excluding adipose tissue as a suitable source for cell therapy. An earlier study demonstrated that injection of osteoblasts differentiated from BM-MSCs induced significantly higher bone formation than those derived from AT-MSCs; however, the cells in that investigation were combined with a polymeric membrane that is known to play a role in the healing process^10^.

In this study, to compare the bone tissue formed by cells with bone already present in the calvaria, termed here as the native bone, nanoindentation assays and gene expression analysis of markers of bone remodeling were conducted. The elastic modulus and the hardness of the newly formed bone induced by BM-MSCs and AT-MSCs were extremely similar to those of the native bone. Mechanical features of bone are strongly correlated to the mineralization status of the tissue within its organic matrix^48,49^. From a biomechanical perspective, the MSCs derived from both sources induced the formation of bone tissue similar to the preexistent calvarial bone before the defect creation.

The molecular signature provided by the gene expression analysis revealed that all the six genes involved in bone formation (*Runx2*, *Sp7*, *Alp*, *Bmp4*, *Oc*, and *Opn*) were upregulated in the bone tissue induced by BM-MSCs compared to that by AT-MSCs, whereas genes involved in bone resorption (*Rankl/Opg* ratio and *Trap*) were upregulated in the AT-MSC-induced bone. Differences in the molecular panel involved in bone formation and resorption may be interpreted as representative of the stage of bone remodeling^50–52^. Such differences in molecular signatures suggest that despite exhibiting similar morphometric measurements, histological characteristics, and mechanical properties, the bone tissue induced by BM-MSCs or AT-MSCs is in distinct stages of the bone remodeling process. Considering the gene expression of native bone as representative of the bone remodeling balance, our results suggest that the bone tissue formed by AT-MSCs, due to its similarity to that of native bone, exhibits a closer equilibrium between formation and resorption. On the other hand, the gene expression data confirm that the bone tissue induced by BM-MSCs is more predominantly in the bone-forming stage.

Although our results have demonstrated significant bone repair in terms of tissue formation and mechanical properties, the regeneration of large bone defects remains a challenge that deserves further investigations. In this context, the use of novel genome-editing tools such as clustered regularly interspaced short palindromic repeats to generate cells with enhanced capacity to modulate the microenvironment could be a smart strategy to promote more bone formation^45,53^. In addition, induced pluripotent stem cells (iPSCs) have emerged as a promising alternative for cell therapy approaches as they exhibit higher proliferation activity, survival rate, and engraftment capacity after transplantation^54,55^.

In conclusion, this study has dmonstrated that cell therapy based on the local injection of BM-MSCs or AT-MSCs can deliver viable cells that induced a significant improvement in bone healing. Despite differences observed in molecular cues between BM-MSCs and AT-MSCs, both were capable of forming bone tissue at comparable amounts and properties. This is a solid step in the field of cell therapy toward the total regeneration of bone tissue in challenging sites, which could be achieved through further investigations using the approach we have demonstrated here in association with cells combined with growth factors or genetically edited cells.

## Supplementary information

Figure S1

## Data Availability

The data used to support the findings of this study are available from the corresponding author upon request.
