# Supplementary material for: Cell Therapy: Effect of Locally Injected Mesenchymal Stromal Cells Derived from Bone Marrow or Adipose Tissue on Bone Regeneration of Rat Calvarial Defects
Source: Sci Rep. 2019 Sep 17;9:13476. doi: 10.1038/s41598-019-50067-6 (PMC6748998; doi:10.1038/s41598-019-50067-6)
Supplement: Supplementary file 1 — Figure S1 [file 41598_2019_50067_MOESM1_ESM.pdf]

Cell Therapy: Effect of Locally Injected Mesenchymal Stromal Cells Derived from Bone Marrow or Adipose Tissue on Bone Regeneration of Rat Calvarial Defects

Gileade P. Freitas,[1] Helena B. Lopes,[1] Alann T. P. Souza,[1] Paula G. F. P. Oliveira,[1] Adriana L. G. Almeida,[1] Lucas E. B. de Souza[2], Paulo G. Coelho,[3,4] Marcio M. Beloti[1], Adalberto L. Rosa[1]

[1] Bone Research Lab, School of Dentistry of Ribeirão Preto, University of São Paulo, SP, Brazil.

[2] Hemotherapy Center of Ribeirão Preto, University of São Paulo, SP, Brazil

[3] Department of Biomaterials, New York University College of Dentistry, New York, NY, USA

[4] Hanjorg Wyss Department of Plastic Surgery, New York University School of Medicine, New York, NY, USA

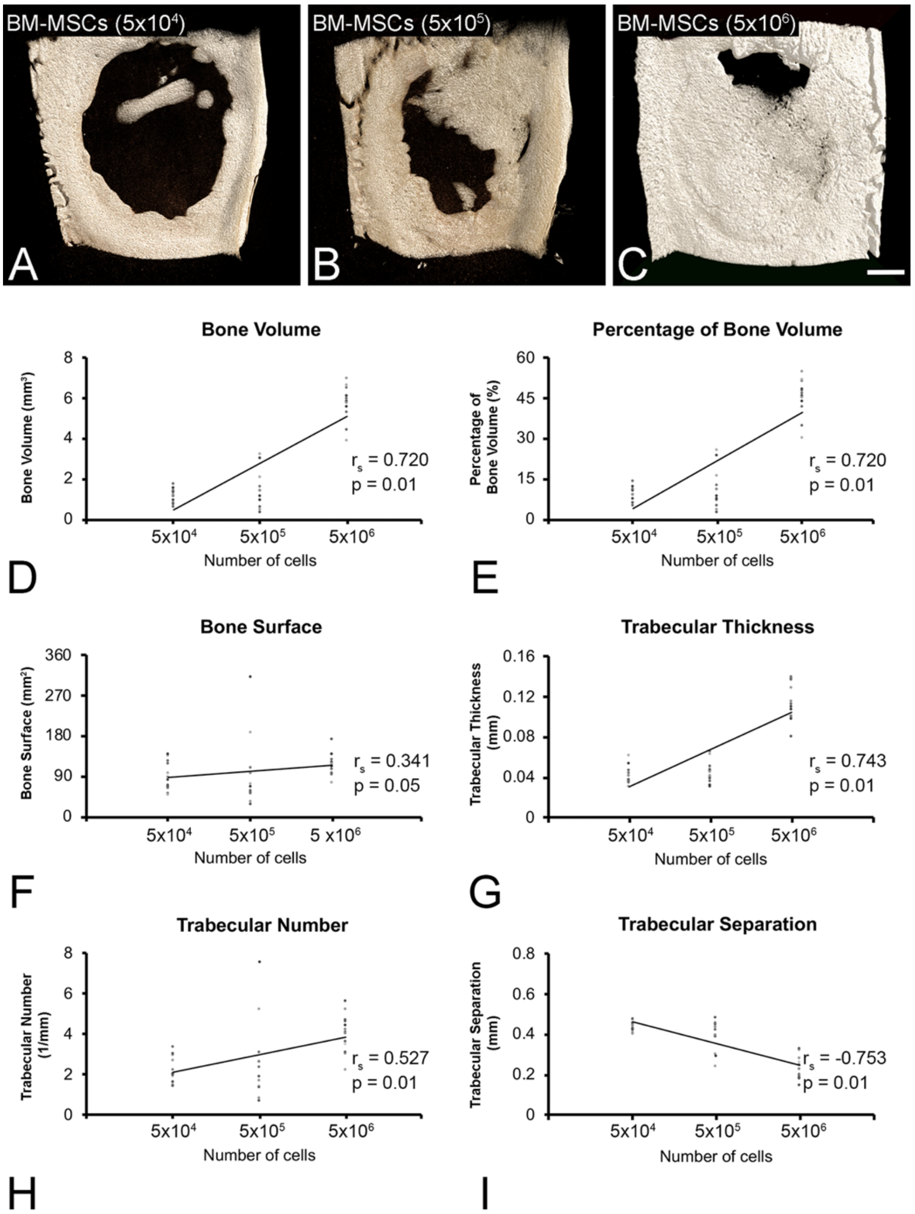

Figure S1. Three-dimensional reconstructed μCT images and morphometric parameters of bone formation in rat calvarial bone defects injected with 5×10^4 bone marrow-derived mesenchymal stromal cells (BM-MSCs, A), 5×10^5 BM-MSCs (B), or 5×10^6 BM-MSCs (C) 4 weeks post-injection (n = 12). Bone volume (D), percentage of bone volume (E), bone surface (F), trabecular thickness (G), and trabecular number (H) exhibited positive correlations, with increases with the increase in the number of injected cells, whereas trabecular separation (I) presented a negative correlation decreasing with the increase in the number of injected cells. Correlation analyses were performed using Spearman's correlation coefficient (n = 12). Scale bar: A–C = 1.25 mm. The data confirm the relationship between the number of cells and bone formation, indicating better results with higher number of cells.
